# Antimicrobial activity of novel symmetrical antimicrobial peptides centered on a hydrophilic motif against resistant clinical isolates: *in vitro* and *in vivo* analyses

**DOI:** 10.1128/spectrum.00265-24

**Published:** 2024-10-09

**Authors:** Chaoqun Zhang, Le Fu, Yuan Zhu, Qigui Chen, Zetong Chen, Yung-Fu Chang, Yide Li, Mengjing Yao, Xinyi Huang, Li Jin, Xue Gao, Yiyu Zhang, Biao Jin, Shuli Chou, Liang Luo

**Affiliations:** 1 Department of Critical Care Medicine, The Seventh Affiliated Hospital, Sun Yat-sen University, Shenzhen, China; 2 Department of School of Medicine, Shenzhen Campus of Sun Yat-sen University, Sun Yat-sen University, Shenzhen, China; 3 Department of Population Medicine and Diagnostic Sciences, College of Veterinary Medicine, Cornell University, Ithaca, New York, USA; University of Saskatchewan, Saskatoon, Saskatchewan, Canada

**Keywords:** antimicrobial peptides, MRSA, antibiotic resistance, symmetric sequence

## Abstract

Antibiotic resistance poses a significant public health threat worldwide. The rise in antibiotic resistance and the sharp decline in effective antibiotics necessitate the development of innovative antibacterial agents. Based on the central symmetric structure of glycine–serine–glycine, combined with tryptophan and arginine, we designed a range of antimicrobial peptides (AMPs) that exhibited broad-spectrum antibacterial activity. Notably, AMP W**5** demonstrated a rapid and effective sterilization against methicillin-resistant *Staphylococcus aureus* (MRSA), displaying both a minimum inhibitory concentration and a minimum bactericidal concentration of 8 µM. Mechanistic studies revealed that AMP W**5** killed bacterial cells by disrupting the cytoplasmic membrane integrity, triggering leakage of cell contents. AMP W**5** also exhibited excellent biocompatibility in both *in vitro* and *in vivo* safety evaluations. AMP W**5** treatment significantly reduced skin bacterial load in our murine skin infection model. In conclusion, we designed a novel centrosymmetric AMP representing a promising medical alternative to conventional antibiotics for treating MRSA infections.

**IMPORTANCE:**

Increasing antibiotic resistance and the paucity of effective antibiotics necessitate innovative antibacterial agents. Methicillin-resistant *Staphylococcus aureus* (MRSA) is a major pathogen causing bacterial infections with high incidence and mortality rates, showing increasing resistance to clinical drugs. Antimicrobial peptides (AMPs) exhibit significant potential as alternatives to traditional antibiotics. This study designed a novel series of AMPs, characterized by a glycine–serine–glycine-centered symmetrical structure, and our results indicated that AMP W5 exhibited a rapid and effective bactericidal effect against MRSA. AMP W5 also demonstrated excellent biocompatibility and a bactericidal mechanism that disrupted membrane integrity, leading to leakage of cellular contents. The notable reduction in skin bacterial load observed in mouse models reinforced the clinical applicability of AMP W5. This study provides a promising solution for addressing the increasing threat of antibiotic-resistant bacteria and heralds new prospects for clinical applications.

## INTRODUCTION

Increasing antibiotic resistance is a significant global public health issue, causing approximately 700,000 deaths annually worldwide due to drug-resistant bacterial infections. It may become more deadly than cancer, with projections indicating approximately 10 million fatalities per year by 2050 (1, 2). *Staphylococcus aureus* is a leading cause of bacterial infections globally. These infections vary from superficial skin and soft tissue infections to severe invasive infections that can result in sepsis and mortality (3). Since their isolation in 1960, the incidence of methicillin-resistant *S. aureus* (MRSA) strains in medical facilities has risen to 25%–50%, with widespread presence in community and animal husbandry settings (4–6). In Europe, MRSA accounts for 44% of healthcare-related infections, 22% of mortality rates, and 41% of additional hospital stays (7). MRSA infections are more complex and costly in healthcare systems (8). Vancomycin has historically been the primary treatment for MRSA; however, the emergence of vancomycin-intermediate and vancomycin-resistant *Staphylococcus aureus* strains has reduced its clinical efficacy (9, 10). Given the emergence of highly resistant organisms, the dire need for new antibiotics and the sharp decline in novel antibacterial agents since the 1990s, developing novel antibacterial agents is imperative (11).

Antimicrobial peptides (AMPs) are low-molecular-weight oligopeptides (< 50 amino acids) characterized by their amphipathic structure, cationic (mean net charge + 3), and hydrophobicity (42%) (12–14). Furthermore, AMPs, as host-defense peptides, are potent antibacterial agents with broad-spectrum activities against various pathogenic fungi, bacteria, and protozoans in plants, animals, and microorganisms. AMPs exhibit high cell selectivity by preferentially targeting anionic bacterial membranes that are rich in phosphatidylglycerol over human cell membranes composed mainly of zwitterionic phospholipids and cholesterol (12–14). Thus, AMPs are considered potential alternatives to traditional antibiotics (15). The AMPs Hylin-a1, PL-5, PMX 300 63, and LTX-10 9 have shown potential efficacy in treating bacterial infections of the skin, nasal cavity, and wounds, including those caused by MRSA (15, 16). Nonetheless, AMPs have some limitations, including heightened cytotoxicity and increased costs, challenges in preserving their bioactive properties and stability *in vivo*, and difficulties in their targeted delivery at therapeutic concentrations to infection sites (1, 17, 18). Therefore, developing safer, more effective, and economical AMPs is necessary.

Previous studies have suggested that the amphiphilic structure of AMPs determines their biological activities. Driven by electrostatic attraction, AMPs initially adhere to negatively charged bacterial membranes via positively charged amino-acid residues. Subsequently, their hydrophobic residues are inserted into the bacterial membrane, causing membrane fragmentation, rupture, and lysis (1, 13, 19). Zhao *et al*. revealed that embedding pairs of tryptophan (Trp, W) facilitated peptide adsorption and insertion into the membrane owing to the strong interaction between Trp and the hydrophobic tails of lipids (20). Additionally, through π–π interaction, Trp promotes peptide aggregation in solution as well as on the surface of the lipid bilayer membrane, aiding conformational changes of peptides and increasing their membrane surface concentration, ultimately enhancing bacterial membrane damage (21). Therefore, Trp was selected as the hydrophobic amino acid in this study. Arginine (Arg, R) was chosen as the hydrophilic charged amino acid due to its ability to induce toroidal pore abnormalities in negatively charged membranes. This may strengthen the electrostatic interaction between peptides and anionic membranes, ultimately enhancing the peptide’s overall charge and its interaction with the negatively charged outer membrane (22, 23). Moreover, previous studies have revealed that a symmetrical structure could help improve cell selectivity and decrease cytotoxicity, regardless of whether the peptide is in the form of α-helices or β-hairpins (24–26). It has been reported that glycine is a low-molecular weight amino acid with short side chains that enhance its activity (27). A previous study has reported that glycine linkers were typically flexible, providing more functional space and extending the flexibility of domains in chimeric or hybrid proteins (28). Additionally, glycine–serine (GS) flexible linkers can improve the stability and biological activity of various fusion proteins without increasing toxicity (29, 30). Therefore, we chose GSG as the center of the symmetric structure.

Considering the above mentioned factors, we designed sequences with a GSG-centered symmetrical structure incorporating both tryptophan and arginine. It has been established that balancing the use of classical Trp and Arg residues is crucial to ensure that the peptides maintain their robust antibacterial activity without excessive cytotoxicity (12, 31). Therefore, based on the symmetric GSG-centered structure, we adjusted the quantity of Arg residues and increased the quantity of Trp residues to achieve an ideal amphiphilic structure. The antibacterial activity, cytotoxicity, and antibacterial mechanism of these novel AMPs were evaluated, and their bactericidal activity and cytotoxicity were also assessed in animal models to uncover their potential clinical applications against MRSA.

## RESULTS AND DISCUSSION

### Peptide design and characterization

**Fig 1:**
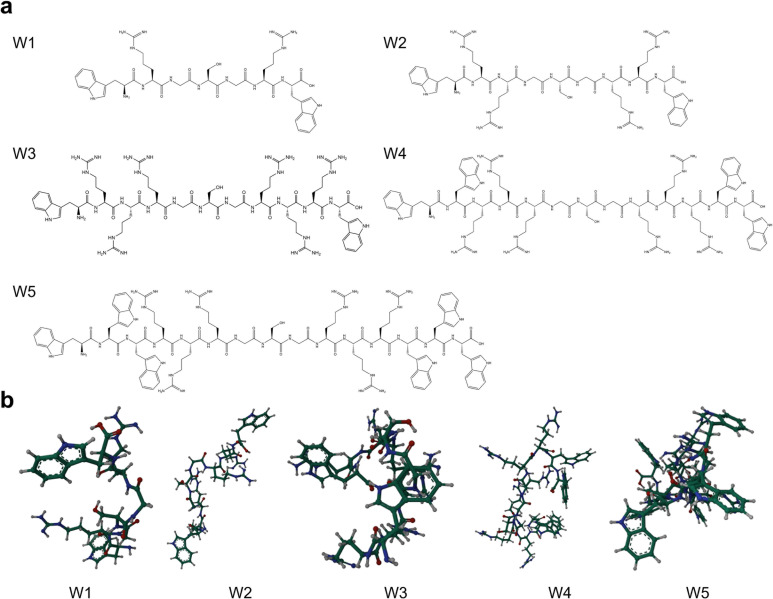
Characteristics of novel designed peptides: (**a**) scheme chemical structural formula; (**b**) three-dimensional structure projections.

Based on the considerations mentioned above, five peptides, designated as AMP W**1**–W**5** were synthesized. The measured molecular weights of the peptides matched the predicted values, confirming the successful synthesis, as validated by MALDI-TOF MS. The structure projections of peptides in two and three dimensions are shown in Fig. 1. The design and key physicochemical parameters of the peptides are presented in Table 1. Peptides W**1**, W**2**, W**3**, W**4**, and W**5** exhibited net charges of 2, 4, 6, 6, and 6, along with hydrophobicity values of 0.13, 0.58, 0.86, 0.43, and 0.11, respectively. The order of amphiphilicity was W**5** > W**4** > W**1** > W**2** > W**3**, with AMP W**5** being the most amphiphilic peptide. Additionally, the peptides exhibit low molecular weights, with the most prominent peptide comprising only 15 amino acid residues, thereby partially alleviating the issue of high costs of AMPs.

**TABLE 1:** Peptide design and their key physicochemical parameters

Peptide	Sequence	Theoretical	Measured	Net charge	Isoelectric point	μHrel^*a*^	H^*b*^	Amphiphilicity index
		Molecular weight (g/mol）	Molecular weight (g/mol）					
W**1**	WRGSGRW-NH2	903	903.02	2	12.1	0.82	0.13	2.68
W**2**	WRRGSGRRW-NH2	1215.37	1215.4	4	12.57	0.52	0.58	2.63
W**3**	WRRRGSGRRRW-NH2	1527.74	1527.78	6	12.8	0.42	0.86	2.6
W**4**	WWRRRGSGRRRWW-NH2	1900.16	1900.16	6	12.8	0.5	0.43	3.26
W**5**	WWWRRRGSGRRRWWW-NH2	2272.58	2272.64	6	12.8	0.19	0.11	3.75

^*a*^ The relative hydrophobic moment (μHrel) of a peptide refers to its hydrophobic moment compared to that of an ideally amphipathic peptide. This comparison provides a more comprehensive understanding of the peptide’s amphipathicity across various scales.

^*b*^ The mean hydrophobicity (H) is calculated by dividing the total hydrophobicity, which is the sum of all residue hydrophobicity indices, by the quantity of residues.

### Antimicrobial assays

**Fig 2:**
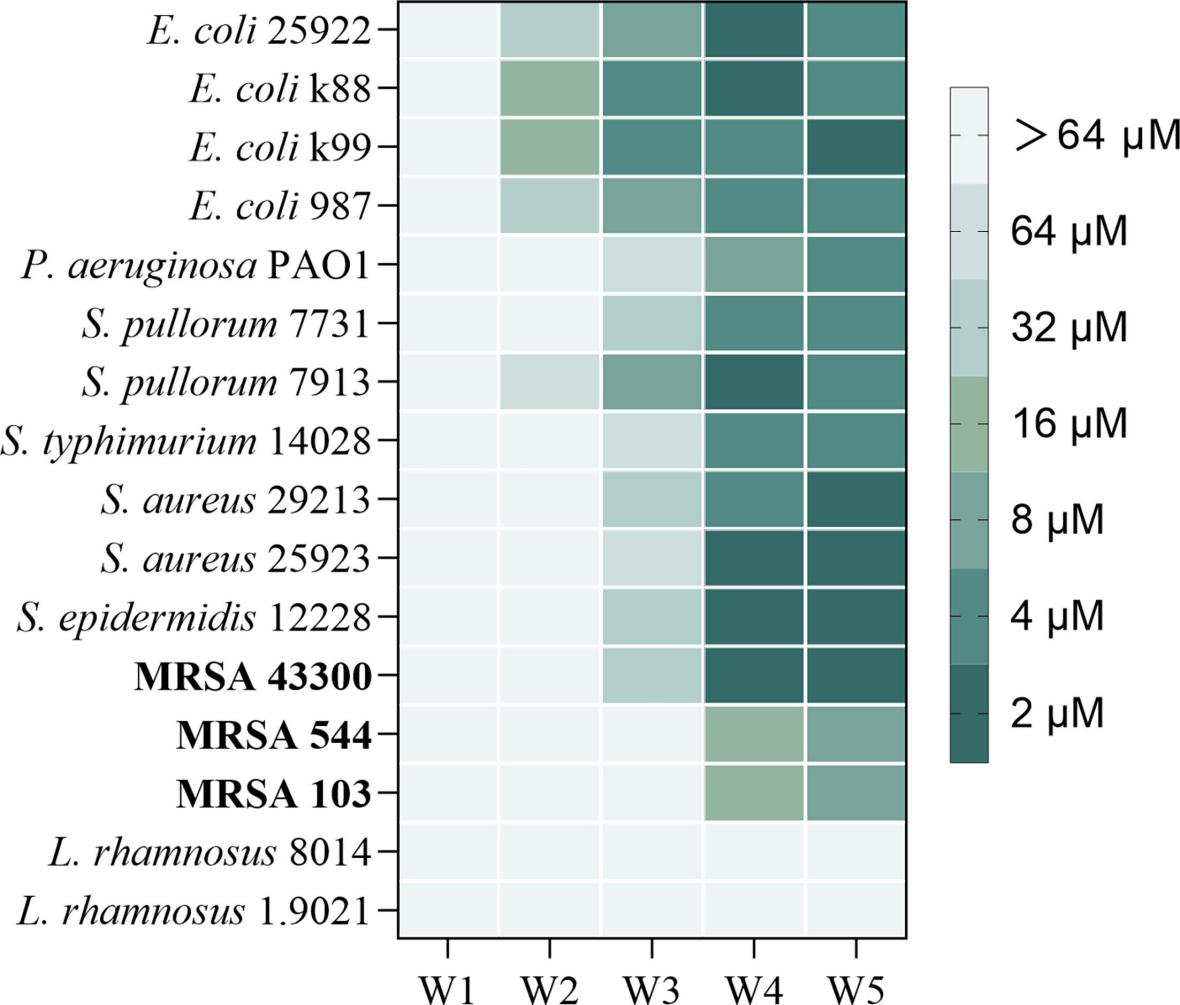
The MIC of the peptides.

The antimicrobial effects of the innovative peptides on typical Gram-negative and Gram-positive bacteria are shown in Fig. 2. AMP W**4** and W**5** were significantly effective against both Gram-negative and Gram-positive bacteria. Notably, AMP W**4** and W**5** exhibited heightened efficacy against *S. aureus*, *Staphylococcus epidermidis*, *Escherichia coli*, and *Pseudomonas aeruginosa*. Furthermore, comparing AMP W**4** and AMP W**5** revealed that AMP W**5** had superior antimicrobial efficiency and specificity against MRSA. Compared to AMP W**4**, AMP W**5** contains a WWW motif, which may account for its high antibacterial activity. Our results corroborate with those of previous studies, which revealed that the WWW motif was essential for eliminating MRSA and destroying preexisting bacterial biofilms (25, 32). Further, AMP W**5** exhibited potent antibacterial efficacy against standard MRSA strains with a minimum inhibitory concentration (MIC) of 2 µM. Despite reducing its antibacterial activity against clinically isolated MRSA strains, which are more resistant to eradication, AMP W**5** still maintained a feasible MIC of 8 µM. These findings underscore the antibacterial potential of AMP W**5**, which displayed noteworthy antibacterial activity against both laboratory-cultivated bacterial strains and clinical MRSA isolates.

To further evaluate the antibacterial efficacy of novel designed peptides against clinically isolated MRSA, we compared their MIC and minimum bactericidal concentration (MBC) values with those of the first-line antibiotic vancomycin and the natural antimicrobial peptide melittin. Vancomycin has long been considered the primary treatment for MRSA infections (9). Melittin (Mel), a naturally occurring peptide with amphipathic properties, has been extensively studied for its pharmaceutical and biological properties. Studies have demonstrated Mel’s efficacy *in vitro* against MRSA, as well as its protective effects in murine models of bacteremia and skin infections. Despite these promising findings, the therapeutic applications of Mel are limited by its significant toxicity and hemolytic impact (33–35). As shown in Table 2, the MICs of clinically isolated MRSA for vancomycin and Mel were 0.5 µM and 4 µM, respectively. Based on the viability measurements after the antimicrobial treatment, the MBC of AMP W**5** against clinically isolated MRSA was found to be equivalent to the MIC, i.e., 8 µM (Table 3). In contrast, the MBC of AMP W**4** for MRSA 544 and MRSA 103 were both 2 × MIC; the MBC of Mel for MRSA 544 and MRSA 103 were MIC and 2 × MIC, respectively; the MBC of vancomycin for MRSA 544 and MRSA 103 were both 8 × MIC. An antimicrobial agent is considered to exhibit bactericidal effects when the ratio of MBC to MIC is less than or equal to 4. Conversely, an agent is categorized as bacteriostatic if the MBC/MIC ratio exceeds 4 (36, 37). Here, the MBC/MIC ratio of AMP W**5** was 1, indicating its bactericidal solid nature against clinically isolated MRSA. Vancomycin, however, only showed antibacterial effects on MRSA 544 and MRSA 103. Compared to traditional antibiotics and classic peptides against Gram-positive bacteria, AMP W**5** still exhibits impressive antibacterial activity targeting clinically isolated MRSA. Thus, AMP W**5** is a promising novel antimicrobial peptide with potential clinical applications against MRSA.

**TABLE 2:** MICs of our peptides and antimicrobial agents against MRSA strains (μM)

	W1	W2	W3	W4	W5	Melittin	Vancomycin
MRSA 544	> 64	> 64	> 64	16	8	4	0.5
MRSA 103	> 64	> 64	> 64	16	8	4	0.5

**TABLE 3:** MBCs of our peptides and antimicrobial agents against MRSA strains (μM)

	W1	W2	W3	W4	W5	Melittin	Vancomycin
MRSA 544	> 64	> 64	> 64	32	8	4	4
MRSA 103	> 64	> 64	> 64	32	8	8	4

Additionally, it is crucial to consider the presence of numerous beneficial probiotics in the human body. Studies have uncovered distinct mechanisms of probiotic action via probiotic-derived functional factors, including immune responses, regulation of intestinal epithelial function, and gut–brain axis (38, 39). However, some specific broad-spectrum antimicrobial agents inhibit the growth of probiotics, thereby diminishing their beneficial effects *in vivo*. In this study, all peptides showed no antibacterial activity against *Lacticaseibacillus rhamnosus* (previously *Lactobacillus rhamnosus*) (MIC > 64 µM), suggesting that they may not hamper the vital role of probiotics in the human body. Therefore, AMP W**5** was further analyzed as a promising antimicrobial peptide.

### Time-kill kinetics study

**Fig 3:**
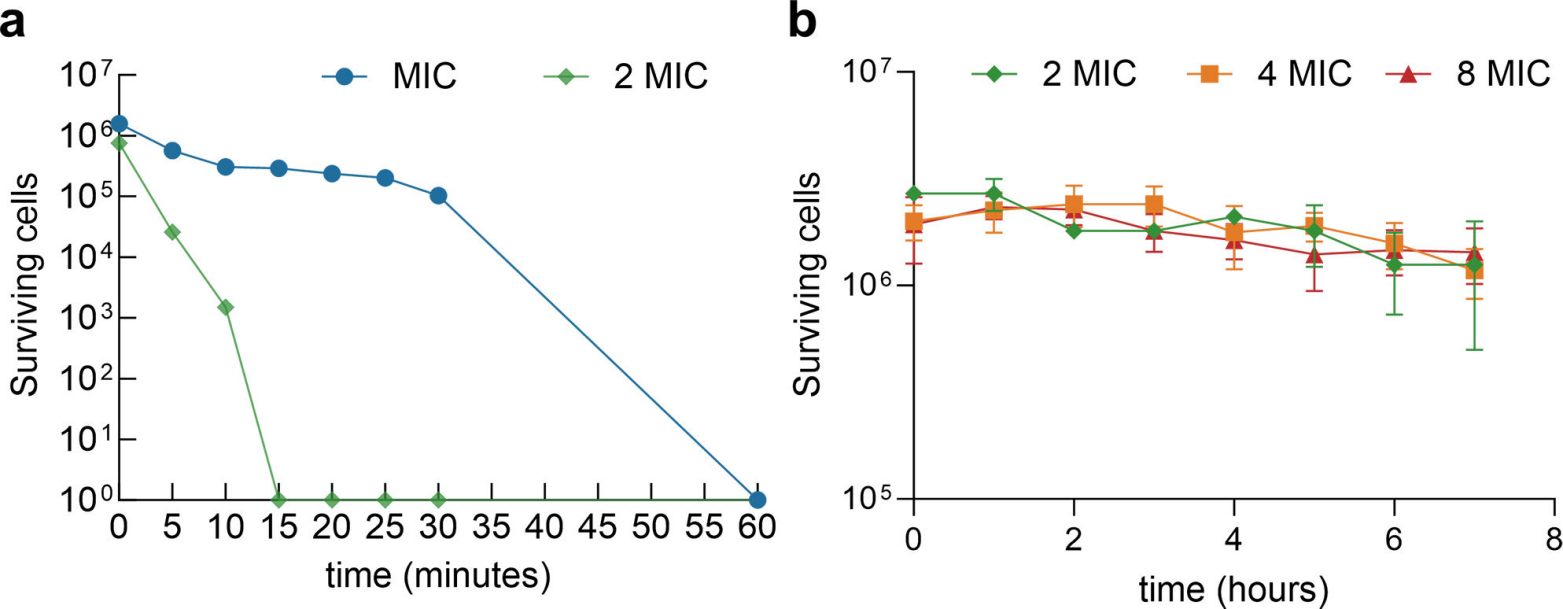
Time-kill curves against MRSA: (**a**) AMP W**5**; (**b**) vancomycin.

A time-kill kinetics assay was used to assess the rapidity of AMP W**5**’s bactericidal action against clinically isolated MRSA. As depicted in Fig. 3a, increasing exposure time to AMP W**5** progressively inhibited MRSA growth. AMP W**5** killed all MRSA cells within 60 minutes at the MIC and 15 minutes at 2 × MIC. Vancomycin, a leading therapy for MRSA infections, was selected as a control for comparative assessment (9). Surprisingly, despite high concentrations of vancomycin (2 × MIC, 4 × MIC, and even 8 × MIC), its bactericidal effect against MRSA was highly sluggish, showing no signs of complete eradication even after 7 hours (Fig. 3b). These results revealed that the rapid bactericidal activity of AMP W**5**, even at relatively low concentrations, underscores its effectiveness in combating MRSA infections.

### Hemolytic analysis of peptides

**Fig 4:**
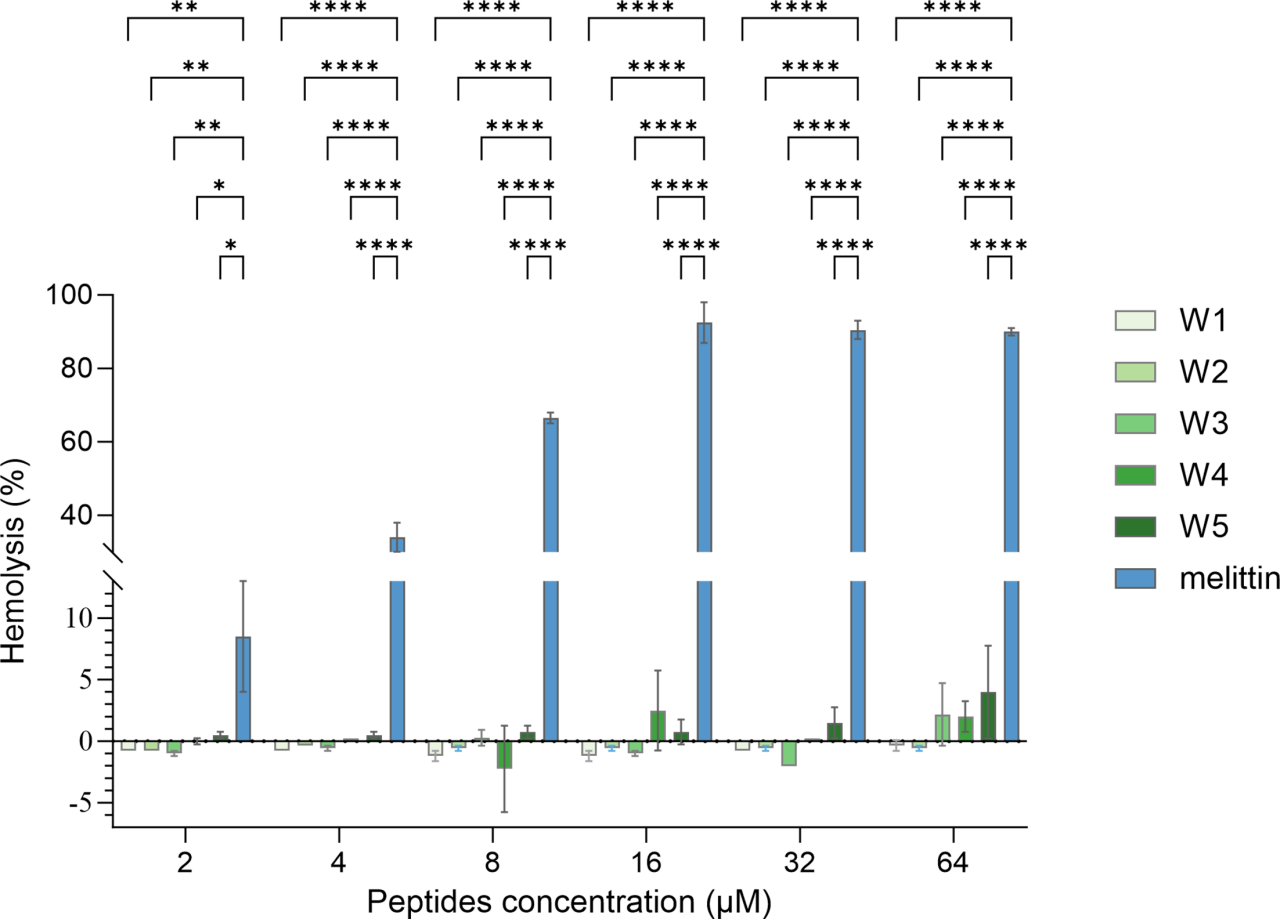
Hemolytic analysis of peptides. Positive control: Triton X-100. Hemolysis (%) = [(Absorbance of sample − Absorbance of PBS) / (Absorbance of Triton − Absorbance of PBS)] ×100. Some negative values, possibly due to experimental variability, and also observed in other studies (41, 45). Statistical significance: *P < 0.05; **P < 0.01; ****P < 0.0001.

Peptide’s impact on hemoglobin release from erythrocyte lysis was evaluated through hemolytic activity assays (40). Studies have revealed that the hemolysis percentage below 10% served as the baseline for the safety and non-toxicity of the peptide (41, 42). As depicted in Fig. 4, various concentrations of AMP W**1**–W**5** (2, 4, 8, 16, 32, and 64 µM) demonstrated minimal hemolytic activity on mouse red blood cells (RBCs) (< 5%). Compared to melittin, all peptides displayed statistically significant differences in hemolytic activity even at low concentrations (≥ 2 µM), indicating that all peptides were safe and non-toxic *in vitro*. As previously mentioned, AMPs usually rely on electrostatic interactions to bind with bacterial cell membranes to exert their antibacterial effects (1, 13, 19). Less-selective AMPs like melittin tend to interact with human cells, ultimately causing significant toxicity and hemolytic effects. Thus, they face limitations in their applications to be used as a control peptide for hemolytic activity (33–35, 41). A symmetrical structure helps improve cell selectivity and decreases cytotoxicity (24–26). Additionally, studies have utilized albumin-binding domain–AMP conjugates and D-amino acid substitutions to reduce cytotoxicity (43, 44). Peptides synthesized in our study possess low molecular weight, a symmetrical structure, and incorporate tryptophan to enhance binding with bacterial membranes, thereby overcoming the hemolytic toxicity while maintaining their superior antimicrobial activity.

### Cytoplasmic membrane depolarization assay

**Fig 5:**
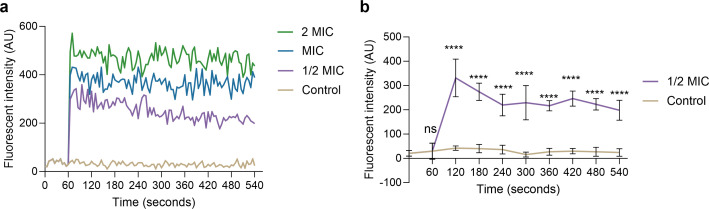
Cytoplasmic membrane depolarization study of AMP W**5** against MRSA: (**a**) Comparison of AMP W**5** at various concentrations versus the control group; (**b**) Significance analysis of AMP W**5** at a concentration of 1/2 × MIC compared to the control group. Control, no peptides. Statistical significance: ns, non-significant, ****P < 0.0001.

Most conventional antibiotics exert antimicrobial effects by interfering with the metabolism and proliferation of microorganisms via site-specific binding mechanisms. However, this approach has a disadvantage: microorganisms can rapidly develop resistance through mutations in binding sites, production of antibiotic-inactivating enzymes, alteration of the cell wall or outer membrane permeabilization barrier, or an antibiotic-activated efflux pump, leading to the loss of drug activity (1, 19, 46). Hence, it is imperative to develop innovative antibacterial agents with mechanisms distinct from those of traditional antibiotics to combat antibiotic resistance. To investigate the potential antibacterial mechanism of AMP W**5**, a cytoplasmic membrane depolarization study was carried out with the potential sensitive fluorescent dye DiSC_3_-5. Typically, DiSC_3_-5 accumulates in the membranes of living bacteria, resulting in low fluorescence intensity due to self-quenching. However, the fluorescence intensity markedly increases when integrity is disrupted. As depicted in Fig. 5, when MRSA was exposed to various concentrations of AMP W**5**, the fluorescence intensity increased dose-dependently. These results demonstrate that the potential bactericidal mechanism of AMP W**5** involves disrupting the bacterial membrane. Consistent with the bactericidal mechanism of most antimicrobial peptides, AMP W**5** inhibits or kills MRSA through the physical disruption of the membrane rather than site specificity, which is advantageous for hindering the generation of new bacteria and the development of drug resistance (1, 19).

### Flow cytometry

**Fig 6:**
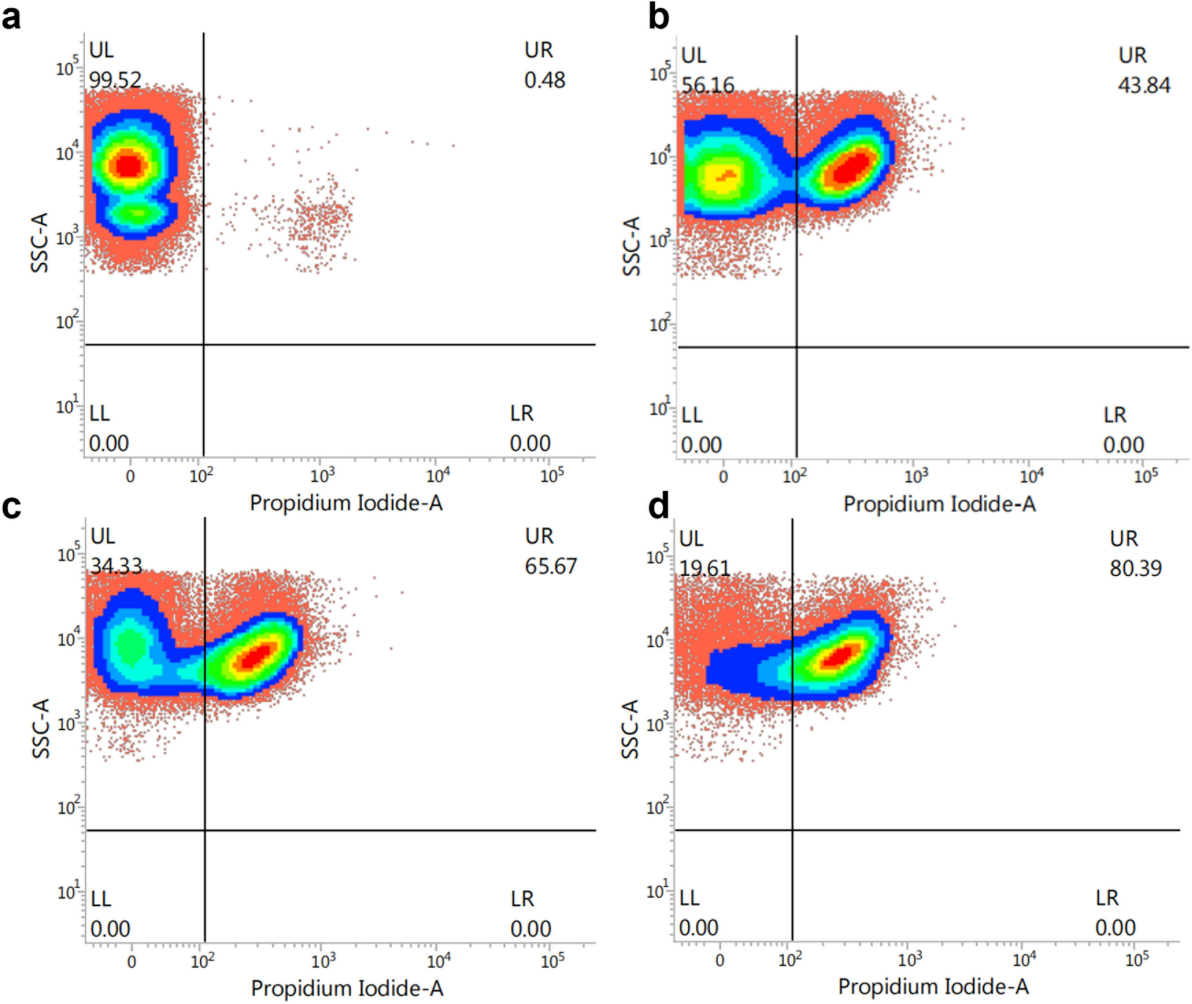
Flow cytometric analysis. MRSA cells were exposed to various concentrations of AMP W**5** for 30 minutes. The increment of the logarithmic fluorescence signal represents the PI uptake generated by peptide processing. The experimental groups included the following: (**a**) Absence of peptide, negative control; (**b**) AMP W**5** at a concentration of 1/2 × MIC (4 µM); (**c**) AMP W**5** at a concentration of 1 × MIC (8 µM); and (**d**) AMP W**5** at a concentration of 2 × MIC (16 µM).

To further determine the mechanism of action of AMP W**5** against MRSA, flow cytometric analysis was employed by propidium iodide (PI) staining, which fluorescently labels nucleic acids in cells with damaged cytoplasmic membranes, serving as a comprehensive measure of cell membrane integrity (47). As shown in Fig. 6, the control group had only 0.48% PI-positive cells, whereas AMP W**5** treatment resulted in 43.84% (1/2 × MIC), 65.67% (1 × MIC), and 80.39% (2 × MIC) PI-positive cells. These results indicated that the addition of AMP W**5** significantly damaged membrane integrity even at a low concentration, further confirming that AMP W**5** killed bacteria by disrupting their cytoplasmic membrane integrity.

### Scanning electron microscopy (SEM)

**Fig 7:**
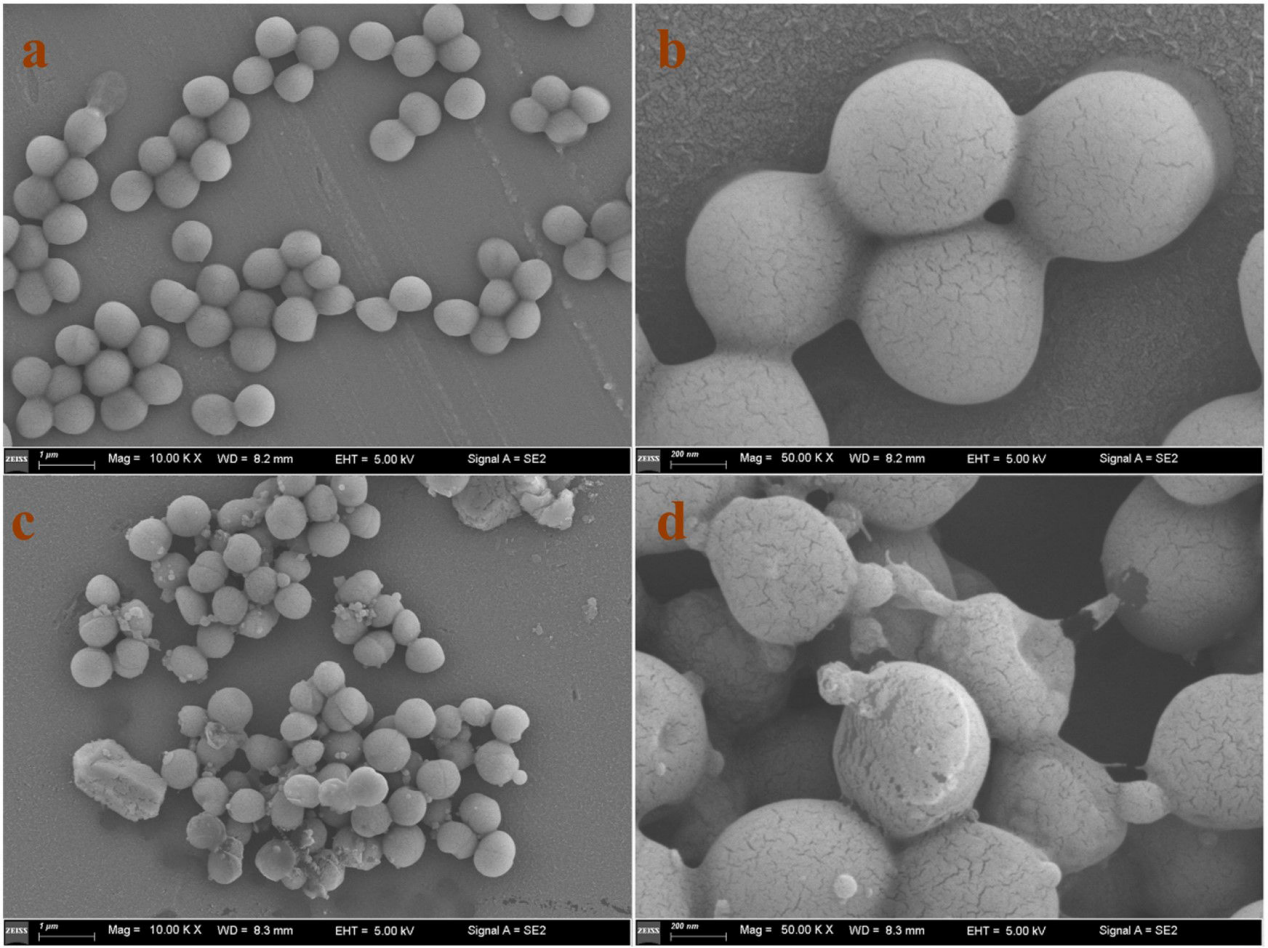
SEM micrographs of MRSA treated with AMP W**5**. SEM micrographs of MRSA: (**a AND b**) Control, no peptides; (**c AND d**) AMP W**5** treated at a concentration of 1 × MIC (8 µM) for 30 minutes. Scale bar = 1 µm (**a, c**) or 200 nm (**b, d**).

To examine the morphological alterations induced by AMP W**5** in bacterial membranes, we employed SEM. The results revealed significant changes in MRSA cell membranes after being treated with AMP W**5**. The untreated MRSA cell membranes appeared smooth and intact, devoid of folds or crevices (Fig. 7a and b). In contrast, after exposure to AMP W**5**, the MRSA cell membranes exhibited noticeable roughening, concavities, and budding structures (Fig. 7c and d). These results further indicated that AMP W**5** exerted antibacterial activity by disrupting cell-membrane integrity.

### Transmission electron microscopy (TEM)

**Fig 8:**
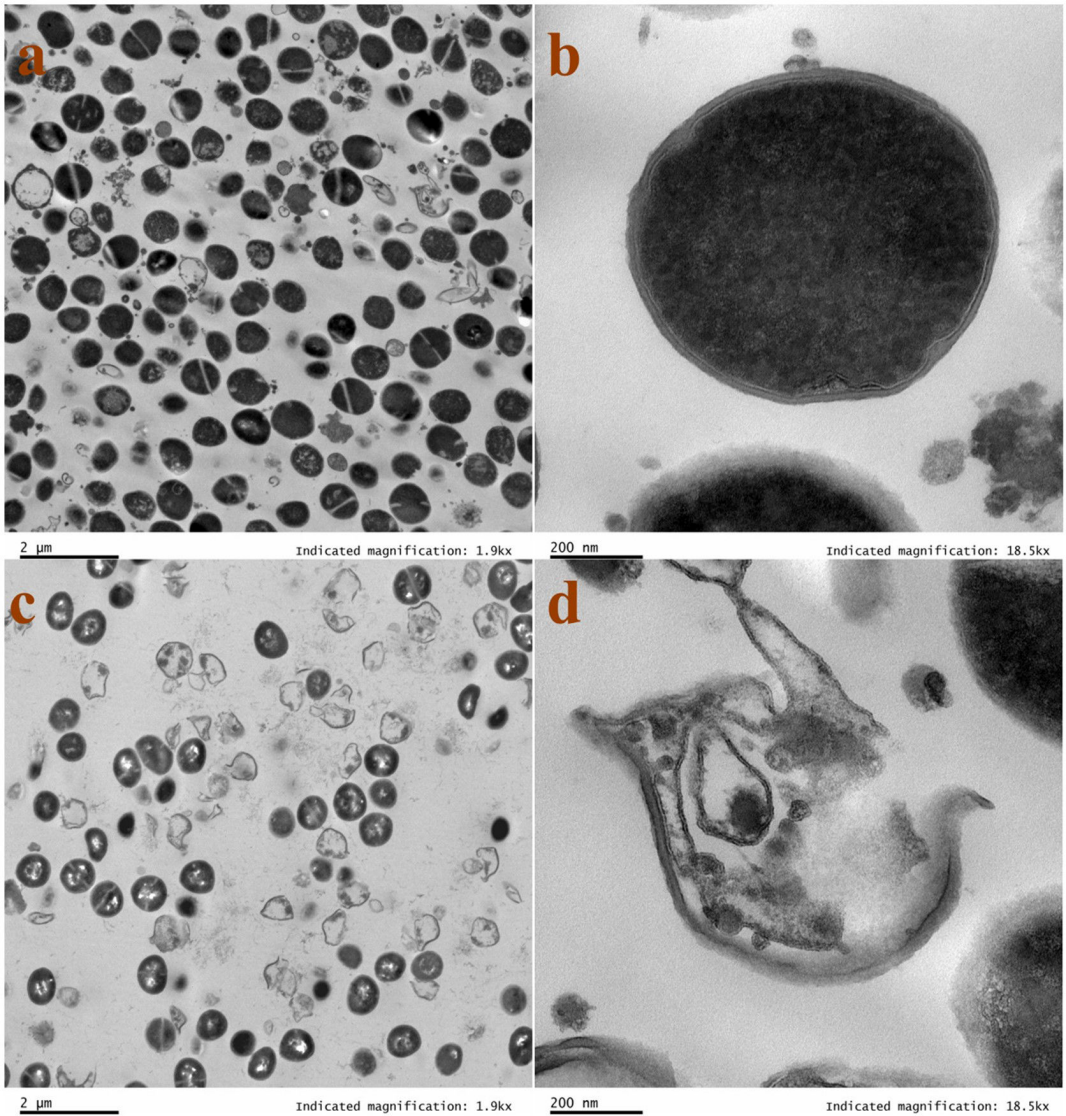
TEM micrographs of MRSA treated with AMP W5. TEM micrographs of MRSA: (a and b) Control, no peptides; (c and d) AMP W**5** treated at a concentration of 1 × MIC (8 µM) for 30 minutes. Scale bar = 2 µm (a, c) or 200 nm (b, d).

Additionally, TEM examinations were employed to delve deeper into the ultrastructure of bacterial cells. The resulting images revealed a stark contrast. W**5**-treated MRSA 544 cells exhibited fragmented cell membranes, evident cytoplasmic leakage, and a hollow interior (Fig. 8c and d), whereas untreated bacteria displayed a smooth and intact surface with a densely packed internal structure (Fig. 8a and b). These observations further underscore the disruptive effect of AMP W**5** on the MRSA cell membranes, leading to significant alterations in bacterial ultrastructure. In addition to membrane-active mechanisms, many AMPs have recently been revealed to target intercellular functions to exert antibacterial activity (48). Certain studies have observed that antibacterial activity was not entirely correlated with membrane disruption or surface morphology. They found that no significant bacterial membrane damage was observed below the antibacterial concentration or sterilization time, but intracellular biomass aggregation was present, demonstrating that intracellular biomass flocculation constituted a pivotal killing mechanism for amphipathic AMPs (49, 50). In our study, the bactericidal kinetics curve showed that AMP W**5** could eliminate MRSA within 60 minutes at 1 x MIC and kill bacteria of the same concentration in just 15 minutes at 2 × MIC. This indicated that AMP W**5** possessed rapid sterilization properties, and the higher the concentration, the faster the sterilization speed. Based on this result, we treated MRSA with 1 × MIC of AMP W**5** for 30 minutes and observed it under TEM. We found that most bacteria showed ruptured cell membranes and loss of intracellular contents, indicating a membrane-disrupting mechanism, consistent with the results of depolarization and flow cytometry, which demonstrated significant cell membrane damage when treated with AMP W**5** even at a lower concentration (1/2 × MIC) (Fig. 5b and 6b). We therefore postulate that membrane disruption is the key mechanism of action for AMP W**5**. However, we noticed a tendency for intracellular biomass flocculation at low magnification under TEM, indicating that this may be an antibacterial mechanism of AMP W**5**. We will continue to investigate this in future studies.

### *In vivo* safety assessment and efficacy evaluation

**Fig 9:**
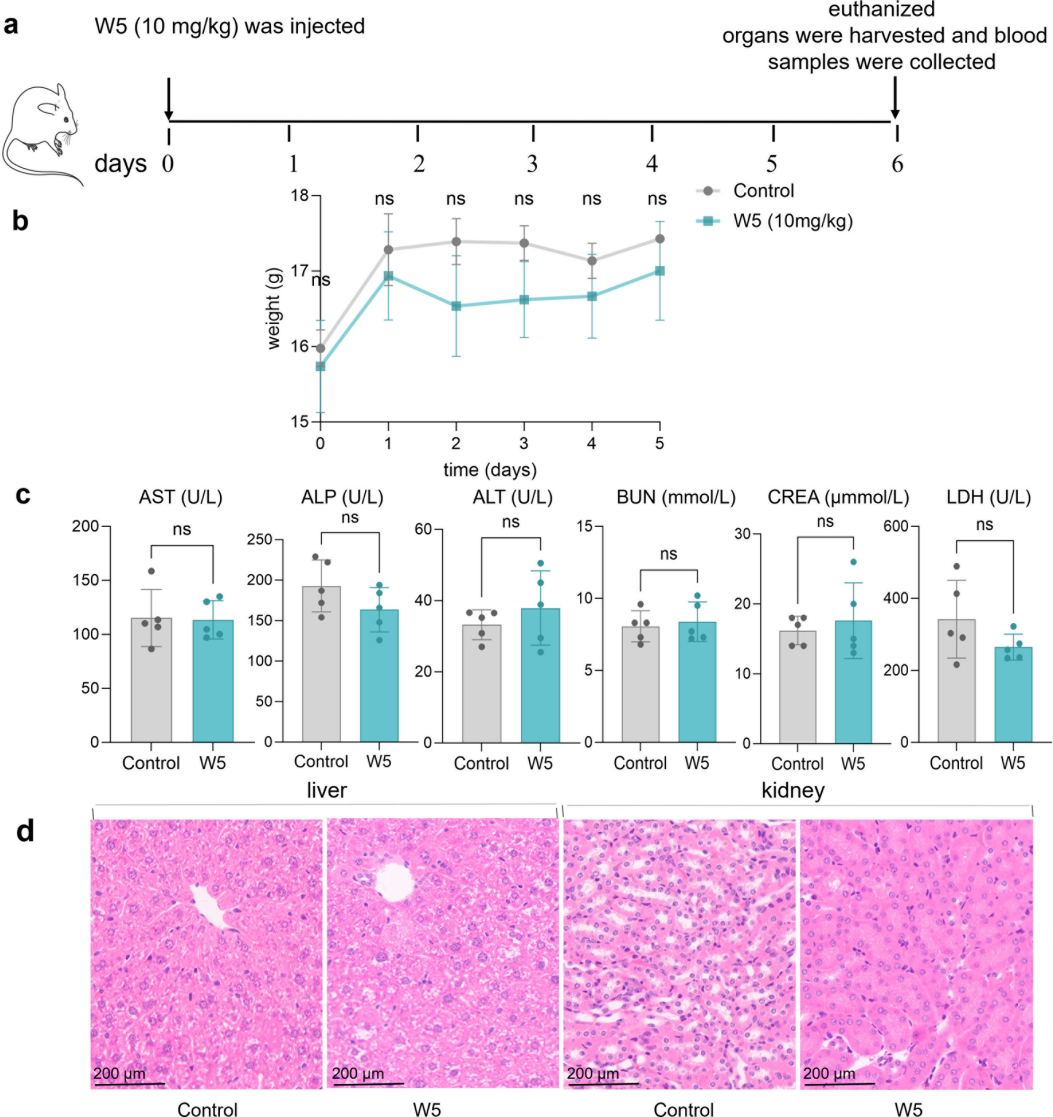
(**a**) Protocol for intravenously injected peptide W**5** to determine *in vivo* safety. (**b**) Weight changes of mice from day 0 to day 5. (**c**) Serum indicator levels of liver and kidney function. (**d**) HE staining of liver and kidney tissues. ns: non-significant. Scale bar = 200 µm.

Based on the potent *in vitro* antibacterial efficacy and high cell selectivity of AMP W**5**, we investigated its potential activity in a murine skin infection model. Initially, 10 mg/kg of AMP W**5** was intravenously injected into mice for *in vivo* safety testing. As illustrated in Fig. 9b, from day 0 to day 5, no statistically significant variation in body weight was observed between the AMP W**5**-treated group and the control group of mice. Additionally, the levels of ALT, AST, ALP, BUN, CREA, and LDH in the serum of both groups remained within the normal range and did not show any statistically significant differences between groups (Fig. 9c), indicating that liver and kidney damages were not observed at the tested concentrations of AMP W**5**. Furthermore, HE staining confirmed that AMP W**5** induced no hepatotoxicity or nephrotoxicity in mice (Fig. 9d). Overall, *in vivo* safety tests demonstrated that AMP W**5** is safe, showing no adverse effects on body weight, liver function, or kidney function, thus overcoming the high toxicity limitations of many AMPs.

**Fig 10:**
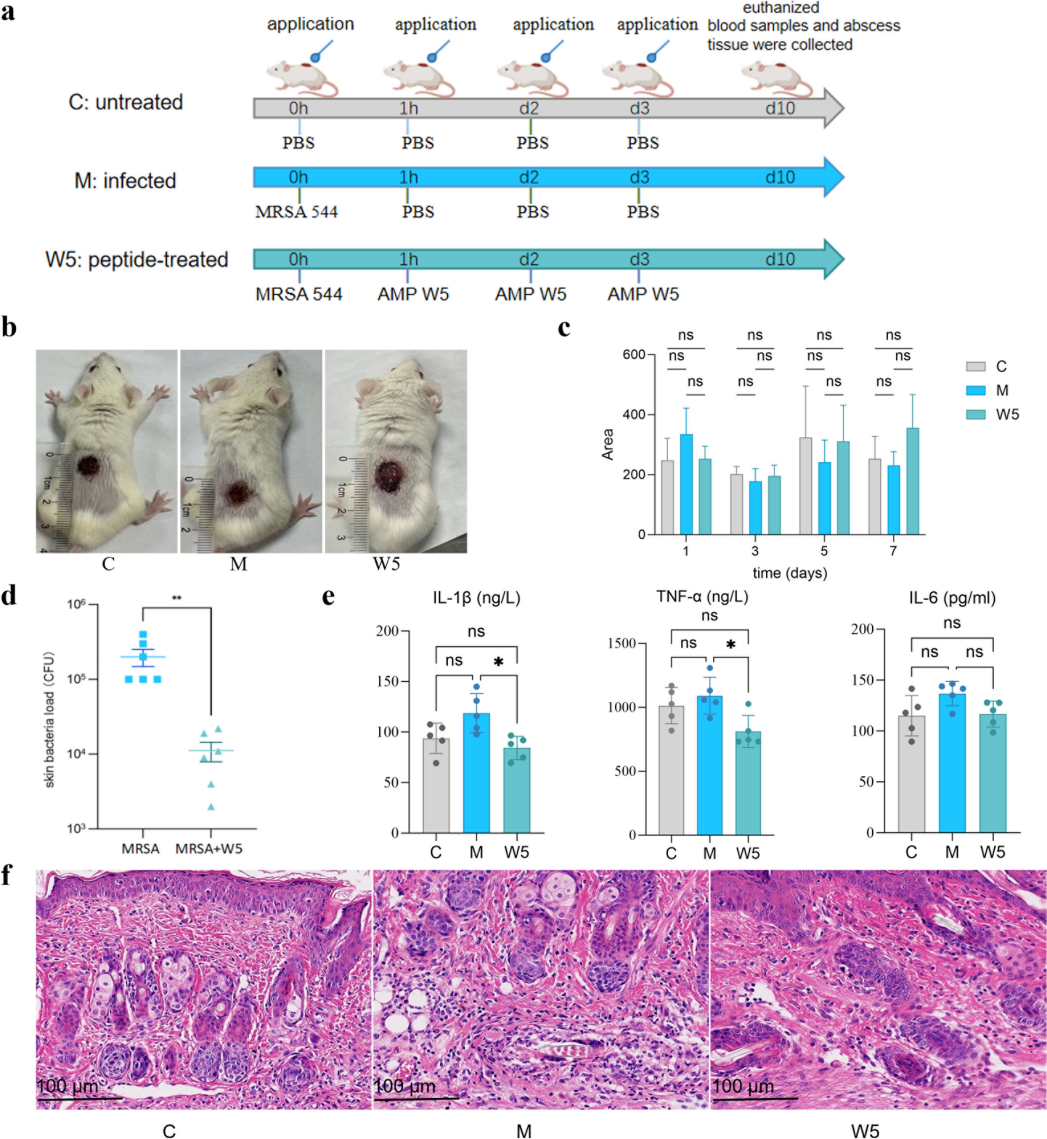
(**a**) A skin infection model protocol to assess the *in vivo* effectiveness of AMP W**5**. (b) Observation of the wound area in mice on day 10. (**c**) Evaluation of the wound area in mice on days 1, 3, 5, and 7. (**d**) The bacterial load *in vivo*. (**e**) Evaluation of proinflammatory cytokines in mice. (**f**) HE staining of mice’s skin and soft tissue. Scale bar = 100 µm. (C, the untreated group of mice without MRSA-infection; M, the MRSA-infected group; W**5**, the peptide-treated group.) Statistical significance: ns, non-significant, *P < 0.05, **P < 0.01.

The primary effects of MRSA are skin and soft tissue infections, accompanied by the release of different virulence factors and proinflammatory cytokines, involving cytolysins, TNF-α, IL-6, IL-33, and IL-1β (51–53). Kim *et al*. have confirmed that cytokines produced by *S. aureus,* particularly MRSA, alter the epidermal lipid composition and consequently cause skin barrier dysfunction (51). Therefore, we established a murine skin infection model to investigate the *in vivo* activity of AMP W**5** against clinically isolated MRSA and its effect on wound healing and proinflammatory cytokines. The results showed that although the skin wound area of AMP W**5-**treated mice increased over time, there were no statistically significant differences among the three groups (Fig. 10b and c). We further explored the bacterial load in the mouse wounds at a microscopic level. As depicted in Fig. 10d through f, after treatment with AMP W**5**, the bacterial load in the skin and soft tissues of the mice decreased. Statistical analysis revealed that the AMP W**5**-treated group exhibited a considerably reduced bacterial load compared to the MRSA-infected group (*P* < 0.01), indicating robust antibacterial activity of AMP W**5** *in vivo*. Additionally, we tested the serum cytokines (IL-1β, IL-6, and TNF-α) in mice. The results demonstrated that the gene expression levels for all cytokines examined consistently ncreased in MRSA-infected mice, in contrast to mice without MRSA infections. After treatment with AMP W**5**, the levels of cytokines in MRSA-infected mice decreased to normal levels (Fig. 10e), implying that AMP W**5** could significantly inhibit the secretion of IL-1β and TNF-α (*P* < 0.05). Briefly, MRSA infections increases proinflammatory cytokines IL-6, IL-1β, and TNF-α gene expression, causing systemic inflammation (54, 55). MRSA-infected mice treated with AMP W**5** may reduce the concentrations of the proinflammatory cytokines, mitigating the systemic inflammatory responses.

Our study adopted a localized application method, applying medication directly to the wounds. The significant reduction in bacterial load at the wound site and the levels of serum cytokines (IL-1β and TNF-α) indicated that the topical medication effectively reached therapeutic concentrations of AMP W**5**, maintaining its antibacterial activity at the infection site and exhibiting certain anti-inflammatory effects, although it did not facilitate wound healing. George Winter demonstrated that moist and permeable wound dressings create an environment more conducive to wound healing, which has been recognized as playing a “pioneering” role in the field of wound healing. Before this, it was believed that keeping the wound dry was best (56). These also suggest that the wound environment plays a vital role in wound healing. Clinical trials have investigated the potential of AMPs for treating infected burns and wounds, but their sensitivity to wound and environmental factors has prevented more comprehensive clinical application (57). In our study, while applying the AMP W5 solution directly to the wound, we encountered challenges in precisely regulating its impact on the wound environment, specifically humidity. This potential influence could have contributed to increased wound area; however, the difference was not statistically significant. Perhaps, subcutaneous administration would not lead to wound expansion. Recently, numerous studies have delved into a range of drug delivery systems, including self-assembly systems, controlled-release hydrogels, nanoparticles, and inorganic materials, all aimed at enhancing the delivery of AMPs to wounds, safeguarding against degradation and extending the biological activity of AMPs (58). In the future, we may further enhance the biological activity of AMP W**5** through these technologies, thereby improving its effect on wounds.

## MATERIALS AND METHODS

### Peptide synthesis

GL Biochem (Shanghai, People’s Republic of China) was used to synthesize and purify the peptides, which were then analyzed for fidelity using MALDI-TOF MS (Linear Scientific Inc., USA). All peptides underwent amidation at the C-terminus to improve structural stability and develop an additional net charge. The peptides were analyzed through reverse-phase high-performance liquid chromatography (RP-HPLC) for purity (> 95%), employing an LC 3000 system (Beijing, People’s Republic of China) with water/acetonitrile 0.1% TFA.

### Peptide characteristics

The physicochemical characteristics of the peptides were determined online (https://dbaasp.org/tools?page=property-calculation). The theoretical molecular masses of the peptides were predicted utilizing an online tool (https://www.novopro.cn/tools/index.html). We used ChemDraw V20.0 or online prediction (https://wemol.wecomput.com/ui/) to draw the scheme chemical structural formula and three-dimensional structures for the peptides.

### Strains

The test strains (Gram-negative: *E. coli* ATCC 25922, *E. coli* k88, *E. coli* k99, *E. coli* 987, *P. aeruginosa* ATCC PAO1, *Salmonella pullorum* ATCC 7731, *S. pullorum* ATCC 7913, and *S. typhimurium* ATCC 14028; Gram-positive: *S. aureus* ATCC 29213, *S. aureus* ATCC 25923, *S. epidermidis* ATCC 12228, MRSA ATCC 43300, MRSA 544, MRSA 103, *L. rhamnosus* ATCC 8014, and *L. rhamnosus* 1.9021) were maintained at the School of Medicine in the Shenzhen Campus of Sun Yat-sen University. Mouse RBCs were extracted from a specific pathogen-free (SPF) grade mouse.

### Antimicrobial assays

The peptides' antimicrobial effectiveness was evaluated using a method derived from a previous publication (59) and modified for use in this study. Single colonies were seeded and cultured in brain–heart infusion (BHI) medium at 37°C until log-phase growth after adjustment to OD_600_ = 0.4, which was followed by a 1000-fold dilution with phosphate-buffered saline (PBS). Then, in sterile 96-well plates, twofold serial dilutions of the peptides were added, followed by 50-µL aliquots of the bacterial cell solution. The ultimate concentrations of the antibacterial agents were 0.125–64 μM. Moreover, bacteria and PBS were added to the TSB as positive and negative controls, respectively. The plates were placed in an incubator set at 37°C for 18 hours. The trial was conducted a minimum of three times in sets of three. Successful completion of the experiment was confirmed by observing the turbidity of the positive control and clarity of the negative control culture medium. The MIC was defined as the lowest peptide concentration required to inhibit bacterial growth.

For MBC analysis, viable cells from all wells with no visible bacterial growth were determined on the BHI agar plates. After 24 hours of cultivation at 37°C, the MBC endpoint was defined as the minimum concentration at which 99.9% of the bacterial population was killed.

### Bactericidal assay

A time-kill kinetics study was carried out by analyzing bacterial survival after exposure to AMP W**5** and vancomycin at different concentrations and durations. MRSA 544 was cultured to the logarithmic phase (OD600 = 0.4) and diluted 1000-fold with PBS. MRSA cells were then treated with AMP W**5** at 1 × or 2 × MIC concentrations. At various periods (0, 5, 10, 15, 20, 25, 30, and 60 minutes), microbial suspensions were serially diluted (10-, 100-, and 1000-fold) with PBS, and 10 µL of the diluted suspension was plated on tryptic soy agar (TSA) medium. Single colonies of microorganisms were counted after 12-hour incubation at 37°C. The processing steps of vancomycin were the same as those of AMP W**5**, but with different concentrations (2 × MIC, 4 × MIC, and 8 × MIC) and periods (0, 1, 2, 3, 4, 5, 6, and 7 hours). Average values were calculated and plotted after six repetitions.

### Hemolytic analysis

Based on a previous study, a hemolysis assay of the peptides was performed using mouse RBCs (21). Briefly, RBCs were centrifuged at 1000 × *g* for 5 minutes, resuspended, and washed thrice with PBS to obtain a 1% (vol/vol) erythrocyte suspension. A 1.5-mL Eppendorf (EP) tube was used to incubate (at 37°C for 1 hour) the erythrocyte suspension (50 µL) with a 50-µL aliquot of serially diluted peptides dissolved in PBS. The concentration of peptides varied from 2 to 64 μM finally. Intact erythrocytes were then centrifuged at 1000 × *g* for 5 minutes at 4°C, and the samples were photographed and observed. Next, 50 µL of the supernatant treated with different peptide concentrations was transferred to a 96-well plate. Hemoglobin release was measured by monitoring the OD at 576 nm. The positive control contains 0.1% Triton X-100, while negative controls comprise PBS. Three replicates of the experiment were conducted, and the percentage of hemolysis was calculated as follows:

Hemolysis (%) = [(Absorbance of sample − Absorbance of PBS) / (Absorbance of Triton − Absorbance of PBS)] ×100

### Depolarization assay

To analyze the interference of AMP W5 on cell membranes, depolarization was determined using DiSC_3_-5 cationic dye. Briefly, log-phase bacteria incubated in Mueller–Hinton Broth (MHB) at 37°C were pelleted, washed with HEPES buffer thrice, and diluted to an OD_600_ of 0.05 using 5 mM HEPES buffer and 0.1 M KCl. Next, cell suspensions were cultured with 0.4 µM DiSC_3_-5 in the dark for 90 minutes at 37°C℃. Various MIC-fold peptides were added, and changes in fluorescence were detected from 0 to 540 seconds by an F-4500 fluorescence spectrophotometer (Hitachi, Japan). The excitation and emission wavelengths were 622 and 670 nm, respectively.

### Flow cytometry

MRSA 544 cells (approximately 10^7^ CFU/mL) were incubated at 37°C for 30 minutes with various concentrations of AMP W5 (1/2 × MIC, 1 × MIC, and 2 × MIC). Subsequently, the bacterial suspension was supplemented with a final concentration of 10 µg/mL PI and left to incubate for an extra 30 minutes. Bacterial cells were collected and suspended in PBS. The FACScan instrument (Becton-Dickinson, San Jose, CA, USA) was utilized for flow cytometry analysis.

### SEM analysis

MRSA 544 cells in the logarithmic phase were diluted to an OD_600_ of 0.2 with PBS and incubated for 30 minutes with AMP W5 at 1 × MIC at 37°C, whereas the control cells were cultured without AMP W5. The cells were centrifuged (5,000 × *g* for 5 minutes), collected, and washed three times with PBS. After overnight fixation with 2.5% (wt/vol) glutaraldehyde at 4°C, the cells were washed three times with PBS for 15 minutes each and post-fixed for 1–2 hours with 1% osmium tetroxide at 4°C. After three additional washes with PBS, the cells were dehydrated in ethanol series (30%, 50%, 70%, 80%, and 90%) for 15 minutes each and, finally, in anhydrous ethanol for 20 minutes each. Subsequently, the cells were transferred to a mixture (vol:vol = 1:1) of anhydrous ethanol and isoamyl acetate for 30 minutes and then incubated for 1 hour with isoamyl acetate. The specimens were dried using the critical point method, coated with gold, and observed using a Zeiss Gemini300 SEM.

### TEM analysis

The sample preparation methods and procedures for pre- and post-fixation were the same as those used for SEM. The cells were dehydrated in a gradient of 50%, 70%, 80%, and 90% ethanol for 10–15 minutes each, in 100% ethanol twice and, finally, in 100% acetone twice, for 10–15 minutes each time. The cells were sequentially transferred into an absolute acetone and an embedding solution mixture (vol:vol = 3:1) for 30 minutes and then to a mixture (vol:vol = 1:1) for 4 hours, before overnight incubation with an absolute embedding solution at 4°C. The specimens were embedded in the embedding plate, baked at 37°C for 24 hours, and then baked at 60°C for 48 hours. The specimens were sliced by using an ultramicrotome, treated with uranyl acetate and lead citrate for staining, and observed through TEM (Tecnai G2 Spirit).

### *In vivo* assays

The *in vivo* safety assessment and antibacterial activity analysis of the peptides were performed using a murine skin infection model wherein 25 SPF-grade 6- to 8-week-old female mice (BALB/c) were randomly divided into five cages (*n* = 5 in each cage). The animals were housed individually in ventilated cages (IVC), at a temperature of 20°C–24°C, humidity of 50%–60%, and a 12-hour day–night cycle.

### *In vivo* safety assessment

Two groups of five mice were randomly divided and intravenously injected with 100 µL PBS or a 10 mg/kg solution of AMP W**5** diluted with PBS. Five days after injection, all animals were euthanized. The weight of each mouse was determined from day 0 to day 5. Blood samples were collected to analyze hepatorenal damage, and an automatic biochemical analyzer was used to measure indicators, including ALT, AST, ALP, BUN, CREA, and LDH. The livers and kidneys of the mice were stained with hematoxylin/eosin (HE) for histological analysis.

### *In vivo* efficacy evaluation

Mice were randomly divided into three (untreated, infected, and peptide-treated) groups of five mice each. After adaptation, hair on the back of the mice was removed, and a 1 cm^2^ hole was punched into the skin on their back. The infected and peptide-treated groups received an application of MRSA 544 (100 µL, 1 × 10^8^ CFU/mL) into the wounds, whereas the untreated group received only PBS. After 1 hour, the peptide-treated group was treated with AMP W5 (100 µL 10 mg/kg), whereas the infected group was treated with PBS for three consecutive days. Wound areas were photographed, and the length (L) and width (W) of the lesion were recorded. The volume of the infected area was calculated as follows:


V=(π /6⋅L⋅W2).

The changes in infection volume were plotted over time. On the 10th day of infection, the mice were euthanized, and blood samples were collected to detect the serum levels of TNF-α, IL-6, and IL-1β using ELISA kits. To analyze the bacterial load, the abscesses were excised, and half of the tissue was diluted with PBS, whereas the other half was fixed with paraformaldehyde. Tissue specimens were stained with HE and independently observed under the light microscope by three investigators.

### Statistical analysis

The mean ± standard deviation is used to present quantitative data. GraphPad Prism version 10.0 was used to conduct significance analysis through one-way analysis of variance, two-way analysis of variance, or the Student’s *t*-test. *P* < 0.05 was considered indicative of statistical significance.

### Conclusions

In this research, we designed a novel series of AMPs characterized by symmetric sequence structures centered around a highly hydrophilic GSG motif. These peptides were engineered with high proportions of Trp and Arg to achieve an optimal amphiphilic balance by adjusting the quantities of these amino acids. The results showed that AMP W**4** and W**5** exhibited broad-spectrum antibacterial activity. Compared to vancomycin, AMP W**5** demonstrated strong bactericidal activity against clinically isolated MRSA and exceptional sterilization efficiency at low concentrations within a short timeframe. Moreover, none of the peptides displayed bactericidal activity against the tested probiotics, suggesting their potential to preserve beneficial bacterial populations and maintain the natural microbial balance in the body. Mechanistic investigations revealed that AMP W**5** exerts its antimicrobial action by disrupting the integrity of cell membranes, leading to cytoplasmic leakage, which likely hinders the development of resistance to AMP W**5**. In animal models, AMP W**5** exhibited safety and efficacy for *in vivo* sterilization. Although there was a perceived enlargement in the wound area among the mice, no statistically significant differences were observed. In addition, AMP W**5** exhibits low molecular weights, both *in vivo* and *in vitro* safety, and antibacterial activity at the site of skin infections, partially alleviating the limitations of high costs, high toxicity, and difficulties in reaching infection sites at therapeutic concentrations. In conclusion, the innovative antimicrobial peptide W**5** presents a promising solution for tackling the growing menace of antibiotic-resistant bacteria, heralding new prospects for clinical applications.

## Data Availability

Data will be made available on request.
